# A Case of Concomitant Emphysematous Cystitis and Clostridium difficile Colitis with Pneumoperitoneum

**DOI:** 10.7759/cureus.2897

**Published:** 2018-06-30

**Authors:** Tooba Tariq, Mehdi Farishta, Asad Rizvi, Furqan B Irfan

**Affiliations:** 1 Medicine, Homer Stryker M.d., School of Medicine/Western Michigan University, Kalamazoo, USA; 2 Medicine, St. Georges University School of Medicine, St. Georges, GRD; 3 College of Osteopathic Medicine, Michigan State University, East Lansing, USA

**Keywords:** emphysematous cystitis, clostridium difficile, colitis, pneumoperitoneum

## Abstract

Emphysematous cystitis (EC) is a rare condition described as air within the wall and lumen of the urinary bladder. It is a complicated form of urinary tract infection caused by gas-forming bacteria. Pneumoperitoneum described as gas in the peritoneum is usually seen with abdominal hollow organ perforation, and approximately 10% of cases have been reported that are not associated with abdominal hollow viscus perforation. To the best of our knowledge, no case of EC with pneumoperitoneum in the setting of concurrent *Klebsiella* urinary tract infection and *Clostridium difficile* (*C. difficile*) colitis have been reported. Here we present a unique case of EC with pneumoperitoneum, in a patient with recurrent *C. difficile* infection and *Klebsiella *pneumonia-urinary tract infection, treated conservatively with a favorable outcome.

## Introduction

Emphysematous cystitis (EC) is a rare condition described as air within the wall and lumen of the urinary bladder. It is a complicated form of urinary tract infection caused by gas-forming bacteria. Historically a finding on autopsy, increasing number of cases have been reported with technological advancement and radiologic imaging. Pneumoperitoneum described as gas in the peritoneum is usually seen with abdominal hollow organ perforation. An uncommon radiographic finding of gas in the peritoneum not associated with abdominal hollow viscus perforation has been reported in approximately 10% of cases with pneumoperitoneum [1]. Both conditions have been managed with observation and supportive care successfully [2-3].

We present a unique case of EC with pneumoperitoneum in a patient with *Clostridium difficile* (C. difficile) infection and *Klebsiella* pneumonia-urinary tract infection treated conservatively with a good outcome. To our knowledge, this is the sixth reported case of pneumoperitoneum secondary to EC and only the second case in which the patient was managed non-operatively without undergoing emergent laparotomy [4-7].

## Case presentation

A 63-year-old woman, nursing home resident, with uncontrolled type-2 diabetes and chronic schizophrenia presented to the hospital with complaints of altered mentation, fever, and profound diarrhea for two days. At the time of admission, she was febrile (temperature 101.5° F) but hemodynamically stable. Pertinent physical exam findings included hyperactive bowel sounds and an abdomen which was tympanitic to percussion. There were no peritoneal signs or rebound tenderness.

**Figure 1:**
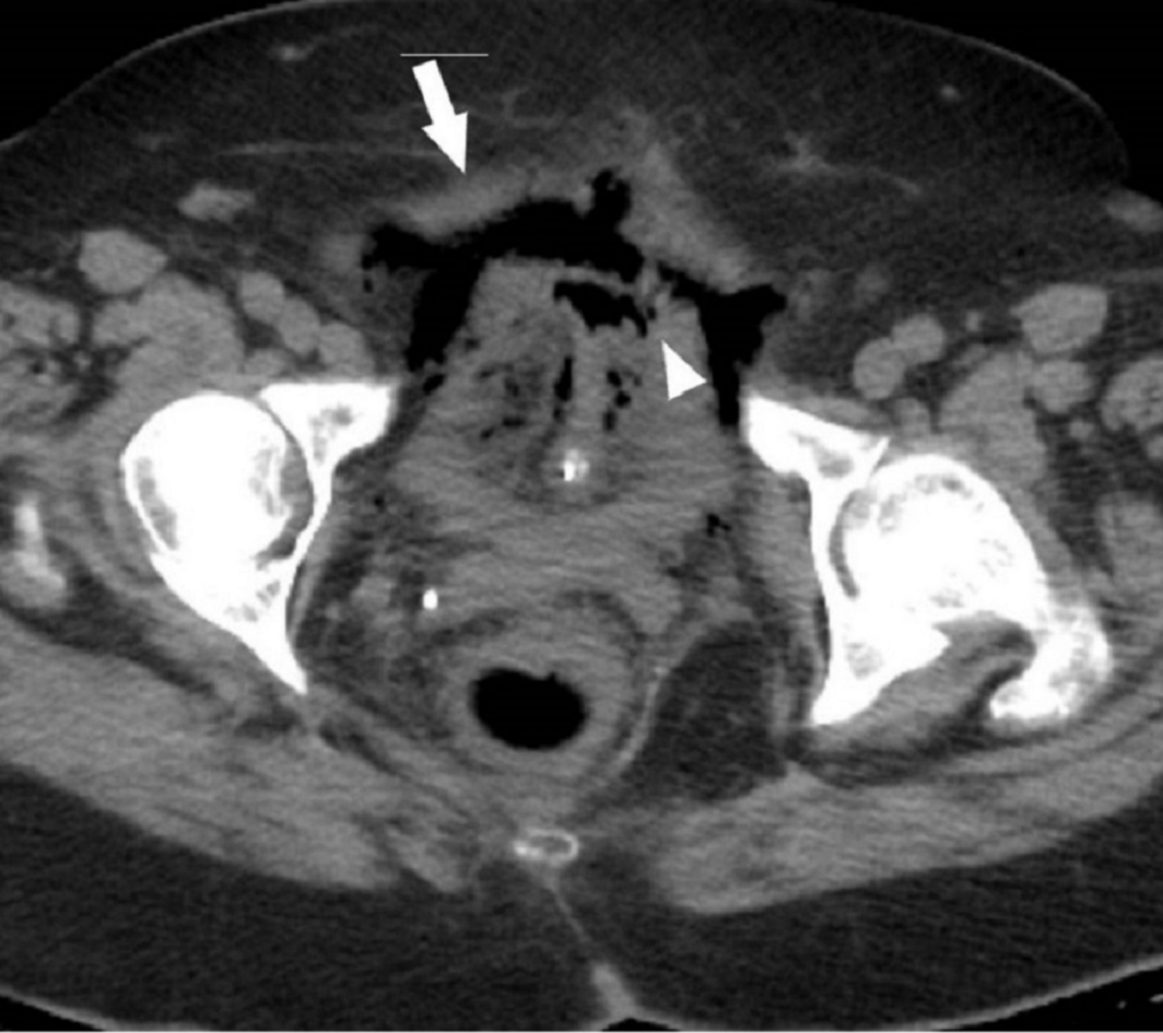
A computed tomographic (CT) scan of the pelvis shows diffuse collection of gas within the bladder wall (arrow head), and air in the peritoneum (arrow)

Initial work-up revealed the following: white blood cell (WBC) count 15,400 cells/mL with 89% neutrophils; serum glucose 195 mg/dL; urea 13 mg/dL; creatinine 1.5 mg/dL; and lactic acid 3 mmol/L. Blood cultures were negative. A urine culture revealed > 100,000 CFU/ml *Klebsiella pneumoniae*. An abdominal computed tomography (CT) scan demonstrated significant (1.6 cm) mucosal wall thickening of the walls of the urinary bladder with perivesical inflammatory stranding. Intraluminal/intramural gas was identified within the walls of the urinary bladder that was consistent with EC with possible perforation (Figure 1). The CT scan further showed pneumoperitoneum predominantly in the anterior pelvis as well as diffuse colitis and proctitis with mesenteric fat stranding. Stool cultures were negative for Salmonella, Campylobacter or Yersinia species. However, the stool polymerase chain reaction tested positive for* C. difficile*.

The patient was intubated due to her low Glasgow Coma Scale (GCS) score and poor airway reflexes. Since the patient did not have signs of acute abdomen or findings requiring emergency laparotomy, she was treated non-operatively. Antibiotics including ceftriaxone were initiated along with oral vancomycin and intravenous metronidazole.

**Figure 2:**
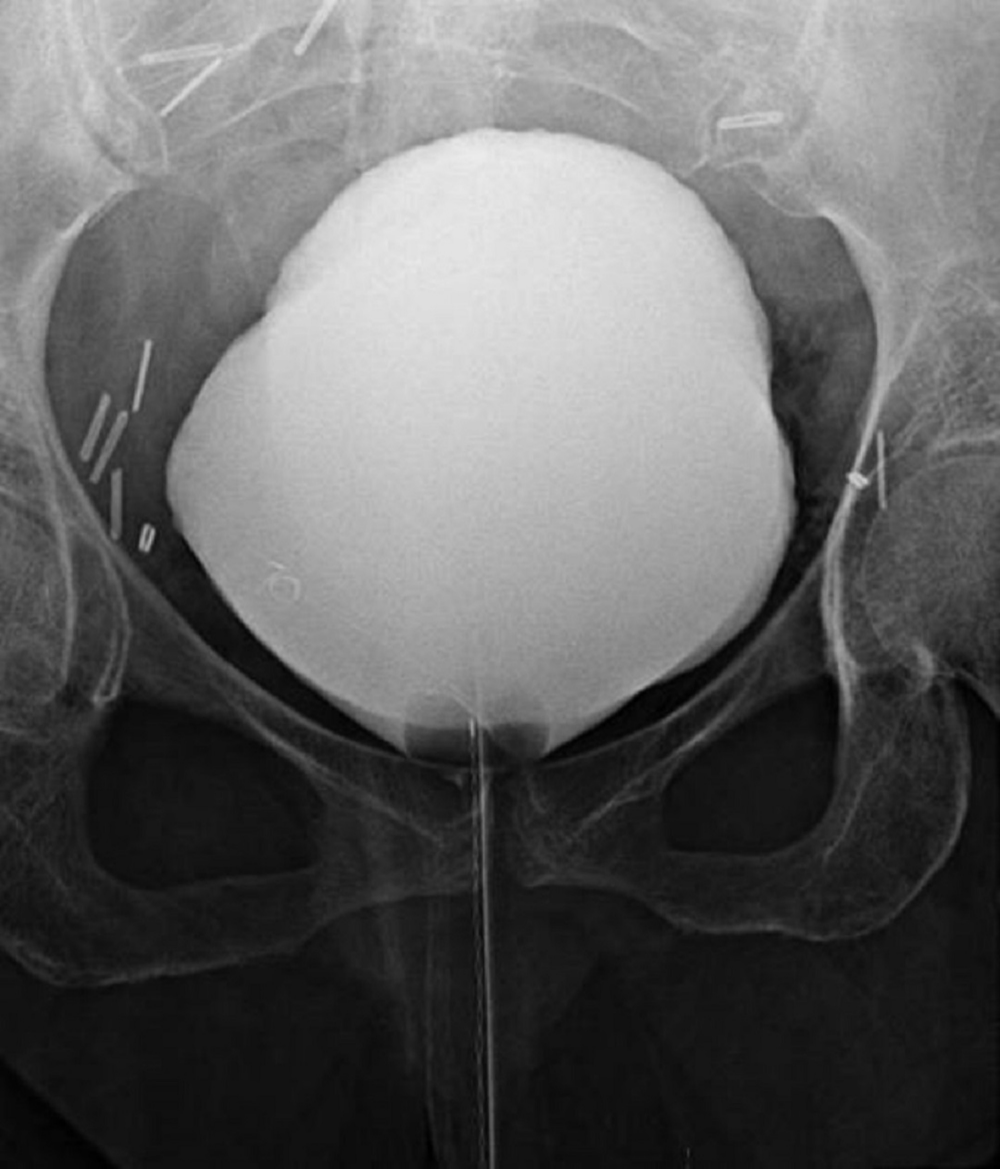
Cystogram showing no evidence of contrast extravasation from the bladder

**Figure 3:**
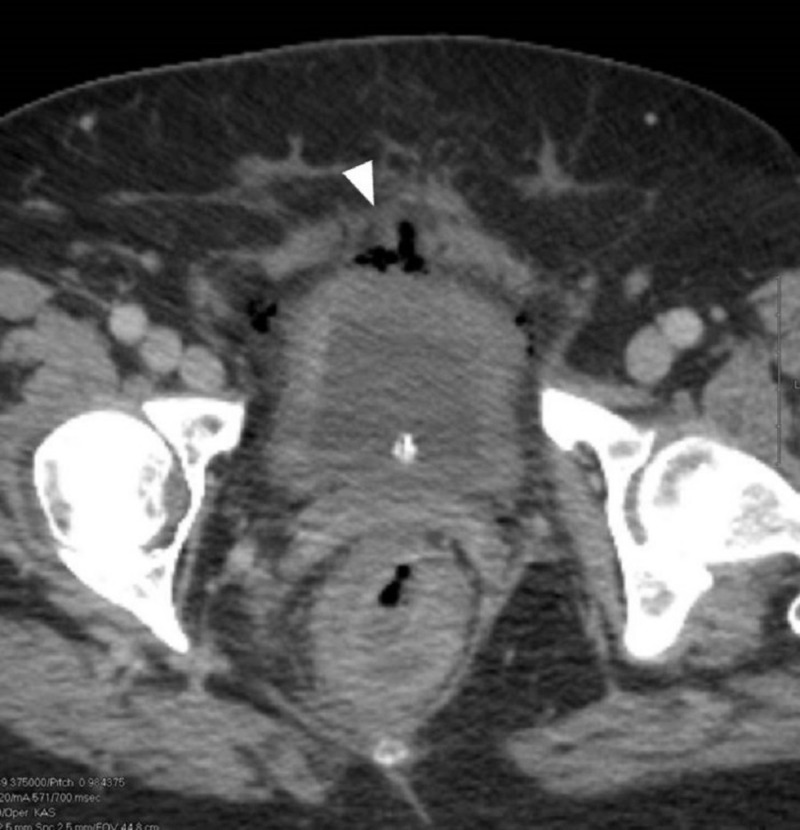
A computed tomographic (CT) scan after two days of therapy shows partial resolution of intra-mural gas from the urinary bladder walls

Urology was consulted and ordered a cystogram to further evaluate for suspected perforation which revealed no radiographic evidence of a bladder perforation and hence recommended conservative management (Figure 2). In consultation with General Surgery for pneumoperitoneum in the setting of diffuse *C. difficile* colitis, it was suspected that the free intraperitoneal air was unlikely secondary to colonic perforation but instead related to EC with tracking of air into the pre-peritoneal space. Conservative management was pursued through the placement of an indwelling catheter for one week. Strict glycemic control was maintained to optimize the patient's uncontrolled diabetes. The patient's fever, diarrhea, and mentation, as well as leukocytosis and renal function, improved and she was subsequently extubated and transferred to the general medical floor on day 4 and discharged to a nursing home on day 12 of hospitalization. A follow-up CT scan three days after discharge showed radiological improvement (Figure 3).

## Discussion

EC is a rare type of urinary tract infection characterized by intraluminal and intramural gas that was first described in detail by Bailey, in 1961. It is typically caused by *Escherichia coli* and *Klebsiella *pneumonia in approximately 80% of cases. Other causative pathogens include *Enterobacter aerogenes, Proteus mirabilis, Staphylococcal aureus, Clostridium perfringes*and *Candida albicans* [8-9].

Some of the well-recognized risk factors implicated in the disease process include female gender, diabetes mellitus, immunocompromised state or neurogenic bladder [3,8]. The patient, in this case, had a history of uncontrolled diabetes mellitus with hemoglobin A1c of greater than 10. The high tissue and urinary concentrations of glucose in diabetics provide a substrate for carbon dioxide production by fermentation [8,10]. This differs from gas forming infections caused by *C. difficile* where the underlying process is thought to be secondary to butyric acid fermentation [11].

The clinical presentations can be highly variable ranging from asymptomatic (7% of the cases) to severe sepsis. The most common symptoms include urgency, frequency, dysuria, pyuria, and hematuria. However, non-specific symptoms such as nausea, vomiting, vague abdominal pain, diarrhea, malaise, fever and altered mentation might be the presenting complaints in a few cases [8]. Pneumaturia is a rare symptom but is quite characteristic of EC [3,12-13].

In our case, the patient presented with fever and altered mentation along with profound diarrhea and was found to have *C. difficile* colitis with findings of extensive left-sided colitis on radiological imaging. There have been studies describing *C. difficile* related pneumatosis intestinalis which is defined as the presence of gas within the intestinal wall. The CT scan of our patient showed no evidence of pneumatoisis intestinalis which has been commonly associated with pneumoperitoneum. Furthermore, there exists sparse data on EC as a complication of severe *C. difficile* colitis [14-15]. The mechanism by which this happens involves pseudomembranous colitis leading to disruption of mucosal barriers with local ischemia eventually giving rise to EC due to the close proximity of the large bowel (particularly the recto-sigmoid colon) and urinary bladder [14].

CT scan is considered the diagnostic modality of choice for EC. A CT scan not only delineates the extent of the disease, whether the infection has spread beyond the bladder to involve the pelvicalyceal systems and renal parenchyma, but is also helpful in detecting any associated air and/or fluid in the peritoneal cavity [16,14], as was demonstrated in our case. The source of the pneumoperitoneum has been attributed to the rupture of intramural blebs in patients with EC, causing tracking of air into the peritoneum without true transmural perforation [15]. In our patient, further confirmation was obtained via cystogram which failed to show any evidence of perforation. Although most cases of EC and pneumoperitoneum can be diagnosed by X-ray or CT scan, in rare cases laparotomy is required to establish the diagnosis [8,14-15].

Management is primarily conservative and includes intravenous antibiotics that target specific pathogens, bladder drainage, and strict glycemic control especially in diabetics. However, in severe cases where medical management is unsuccessful or there is evidence of secondary spread or necrosis, a partial cystectomy/ cystectomy or surgical debridement is warranted [9,17]. Urgent laparotomy may be needed in case of findings suggestive of acute abdomen along with radiographic evidence of air within the peritoneum. Chong et al. and Keisuke et al. reported cases of EC that underwent exploratory laparotomy due to acute abdominal pain in conjunction with peritoneal signs and gas accumulation in the peritoneal cavity [8,14].

Although historically perceived as a grave diagnosis with surgery as the only available cure, EC now has a better prognosis with reported death rates of around 5%-10%. In comparison, emphysematous pyelonephritis is associated with a significant mortality rate (approximately 50%) [8,12]. Impaired renal function and hematuria are predictors of poor outcome for patients with emphysematous pyelonephritis but not for patients with EC [18].

## Conclusions

We report a case of EC with pneumoperitoneum, which responded successfully to non-operative management. Pneumoperitoneum secondary to EC is an extremely rare finding. Also, there is data suggesting that *C. difficile* infection might be associated with the occurrence of EC; however, in our case *Klebsiella* urinary tract infection was the most likely cause. Patients with EC related pneumoperitoneum without signs of acute abdomen and fewer comorbidities should respond favorably to conservative management without the need for exploratory laparotomy or other surgical interventions.
